# Cost-Effectiveness of Day Surgery With Remote Patient Monitoring for Acute Cholecystitis: Economic Modeling Study

**DOI:** 10.2196/76807

**Published:** 2025-10-20

**Authors:** John Paul Kuwornu, David Brain, Kheng-Seong Ng, Amina Tariq, Melissa Baysari, Sundresan Naicker, Adeola Bamgboje-Ayodele, Adrian Boscolo, Peter J Lee, Steven M McPhail

**Affiliations:** 1 Australian Centre for Health Services Innovation (AusHSI), School of Public Health and Social Work, Centre for Healthcare Transformation, Faculty of Health, Queensland University of Technology, Level 7, 88 Musk Avenue, Kelvin Grove QLD, Brisbane, Queensland, 4059, Australia, +61 7 3138 6115; 2 Concord Institute of Academic Surgery, Concord Repatriation General Hospital, Concord, New South Wales, Australia; 3 Susan Wakil School of Nursing and Midwifery, Sydney School of Nursing, Faculty of Medicine and Health, The University of Sydney, Camperdown, New South Wales, Australia; 4 Discipline of Design, School of Architecture, Design and Planning, The University of Sydney, Camperdown, New South Wales, Australia; 5 Sydney Local Health District, Sydney, New South Wales, Australia; 6 Department of Colorectal Surgery, Royal Prince Alfred Hospital, Sydney, New South Wales, Australia; 7 Digital Health and Informatics Directorate, Metro South Health, Brisbane, Queensland, Australia

**Keywords:** digital health, virtual care, economic evaluation, hospital, costs, laparoscopic surgery

## Abstract

**Background:**

Reducing the time to surgery for patients requiring cholecystectomy may lessen the risk of adverse outcomes. Dedicated day-surgery lists supported by out-of-hospital remote monitoring have been explored as a potential solution; however, the cost-effectiveness of such innovative care models remains largely unexplored.

**Objective:**

This study presents a cost-effectiveness analysis comparing an acute day-surgery care model with remote patient monitoring to a conventional inpatient-centric care model for high-acuity cases of cholecystitis.

**Methods:**

Post-surgical complications, effectiveness (measured by bed days saved and quality-adjusted life years [QALYs]), and health care costs associated with the two models of care were compared over a 1-year time horizon using a decision tree model. Health care costs were estimated from the Australian health care funder perspective and expressed in 2023 Australian dollars. Uncertainty was assessed using both deterministic and probabilistic sensitivity analyses.

**Results:**

The acute day-surgery care model dominated the conventional inpatient-centric care model by saving a mean of 1.7 inpatient days per patient (3.2 days for the conventional model versus 1.5 days for the acute day-surgery model) and lowering net health care costs by a mean of AU $1,416 (US $935) per case over the 1-year time horizon. There was no meaningful difference in QALYs between the care models. These results remained robust in both deterministic and probabilistic sensitivity analyses.

**Conclusions:**

An acute day-surgery care model with remote patient monitoring for individuals with acute cases of cholecystitis requiring cholecystectomy would likely free bed days and provide economic benefits to the health care system compared to inpatient-centric practice. Uncertainty in QALY estimates remains a limitation.

## Introduction

The effective management of surgical caseloads and theater resourcing can be challenging in high-demand hospital environments [1,2]. This is further complicated by critical care and surgical ward bed availability constraints [2,3]. International guidelines and local policies advocate for the prioritization of emergent surgical cases to preserve life and support patient safety [1-3]. Consequently, less urgent procedures may be postponed, which may be associated with an increased risk of adverse outcomes in some cases [1-5].

Cholecystectomy as an effective treatment for people with acute cholecystitis who have presented to hospital emergency departments (EDs) is an important case in point [3-6]. Evidence from meta-analyses indicates that avoidable delay in time to surgery for patients requiring cholecystectomy may be associated with a greater risk of adverse outcomes and inefficient hospital resource use [4-6]. After presenting to the ED, patients with less urgent cases may initially be scheduled for next-day cholecystectomy procedures and remain as inpatients until the procedure and recovery are complete. However, for patients who have presented to hospitals that have large emergency caseloads, there is a significant and predictable risk that patients’ cholecystectomy procedures will be de-prioritized relative to higher acuity cases, resulting in long lengths of stay in the hospital [3-6].

A potential solution that has been proposed is the dedicated day-surgery lists for low-risk cases, supported by out-of-hospital care, including the potential for remote monitoring through integrated virtual care or hospital-in-the-home care models [7-9]. The evidence around such novel care models is emerging, including meta-analyses that have indicated virtual wards and hospital-in-the-home care models can produce similar or potentially better outcomes for patients than conventional hospital inpatient care [7-9]. This innovative solution may have particular relevance for EDs with large critical and urgent caseloads, as well as multiple-theater arrays that are not fully used due to costs associated with labor resourcing for operating theaters. In these facilities, through allocation of additional resourcing for dedicated theater lists, there is potential for less urgent patients requiring cholecystectomy to return home after their initial presentation to the ED and present the following day for prompt planned day-surgery with the intention of returning home with remote patient monitoring and support, minimizing their overall inpatient stay.

While care models of this nature have the potential to reduce the length of stay and improve efficiency in resource use without negatively impacting patient outcomes [7-9], their cost-effectiveness remains largely unexplored. Cost-effectiveness is an important consideration for these cases, as a viable economic case must typically be made for the allocation of additional resourcing to establish dedicated day surgery lists for low-risk procedures of this nature. The aim of the present study was to estimate the cost-effectiveness of an acute day-surgery model of care, which may also be known as a same-day discharge model of care, with remote patient monitoring support in comparison to a conventional inpatient-centric model of care for acute cases of cholecystitis.

## Methods

### Scope of the Clinical Population

The population for the base case analysis comprised adult patients presenting to an ED with benign gallbladder disease for which cholecystectomy would typically be indicated on the same admission, and who were considered to have low surgical risk. Recently, based on evidence synthesis, Rickward et al [10] developed an “optimal” inclusion criterion for successful same-day cholecystectomies, which contributed to informing our base case. For example, the inclusion criterion considered patients younger than 65 years, American Society of Anaesthesiologists (ASA) physical status classification of 1 or 2, those with no prior upper abdominal surgeries, those with no or low risk for common bile duct stones, and those who had a responsible adult at home. The majority of biliary pathologies would include acute or chronic cholecystitis and intractable biliary colic [11]. The model assumed patients first presented to a hospital ED with a large emergency caseload within 24 hours of acute pain onset, and that diagnosis was confirmed by abdominal imaging. The following patient-type exclusions were considered out-of-scope for the modeling: previous receipt of cholecystectomy for the treatment of neoplasms, cholecystectomy concurrent with another procedure, unsuitable for minimally invasive surgical intervention or where open procedures would be planned from the outset [11].

### Treatment Strategies

**Figure 1.**
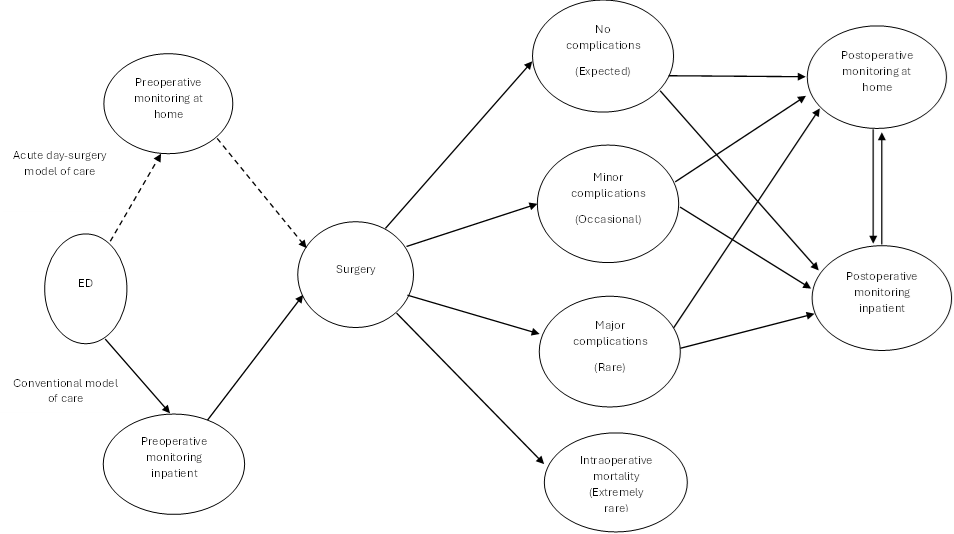
Patient clinical pathways and surgical outcome states under the new acute day-surgery model of care contrasted with the conventional model of care. ED: emergency department.

Our study compared two treatment strategies from the Australian health care funder perspective [1]: (1) a new acute day-surgery model of care with remote patient monitoring, and (2) a conventional inpatient-centric model of care. We adopted this perspective to inform future health care policy from the perspective of health care funders deciding whether to implement a dedicated surgical list for cholecystectomies. Under the conventional model of care, the patient is admitted as an inpatient after presentation to the ED with acute cholecystitis. The patient remains an inpatient until cholecystectomy is performed after allowing for potential delays due to emergency surgical cases consistent with hospitals that have large emergency caseloads. This contrasted with the new acute day-surgery model of care in which patients received initial assessment and surgical work-up in ED then returned home remaining under remote patient monitoring while a cholecystectomy is scheduled within 24 hours of the initial presentation on a dedicated ‘same-day discharge’ surgical list, on which other appropriate same-day acute cases (such as abscess drainages) would be operated on as same-day hospital admissions [12]. Under both treatment strategies, the surgical outcome determined the length of recovery time the patient spends post-surgery in the hospital. Under the new model of care, patients who experienced no complications post-surgery are placed on an expedited discharge protocol supported by remote patient monitoring, while those with post-surgical complications follow the usual care post-surgical inpatient protocol. In both care models, cholecystectomies could be performed laparoscopically or converted to open at the surgeon’s discretion [13]. We focused on laparoscopic cholecystectomy and converted to open cholecystectomy procedures in this study because they account for approximately 95% of all cholecystectomies in Australia [10]. Figure 1 illustrates potential clinical pathways under the two models of care.

### Decision Model

**Figure 2.**
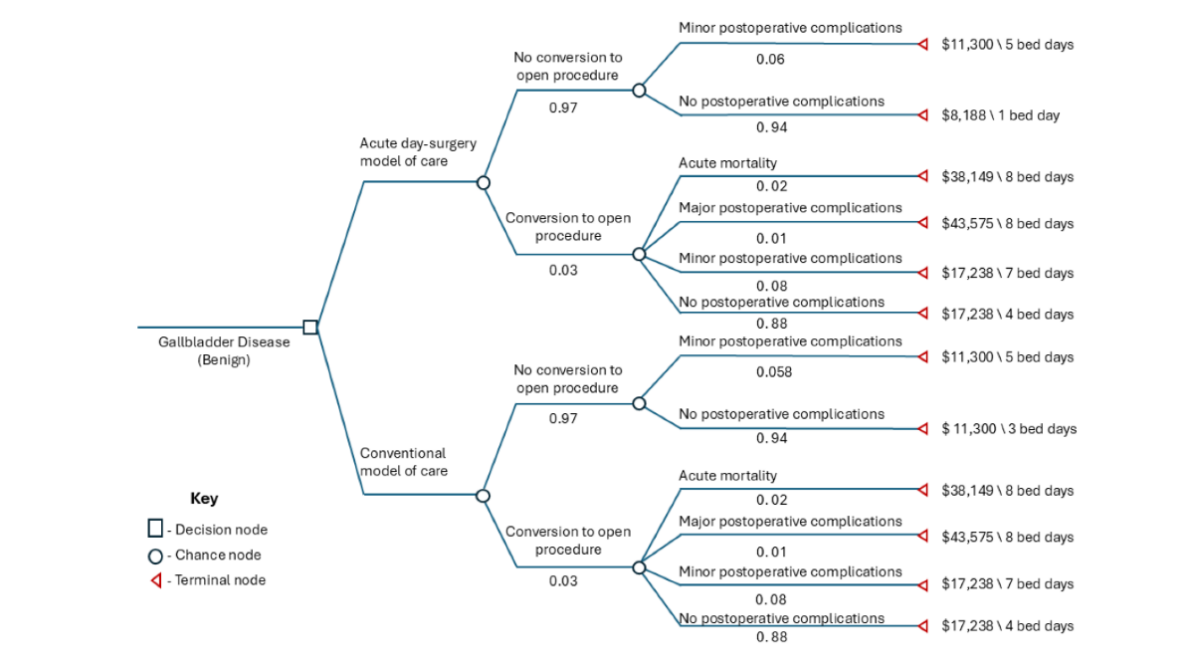
The decision tree model structure. In decision trees, each node type represents a specific function in the decision-making process. A decision node (square) indicates a point where a choice between different strategies or actions is required. A chance node (circle) represents uncertainty, with outcomes determined by assigned probabilities. A terminal node (triangle) marks the end of a pathway, where model payoffs for patients reaching this outcome are summarized*.*

Using decision analysis software (TreeAge Pro, Williamstown, MA), a decision tree model (Figure 2) was created to analyze and compare the costs and outcomes of the two models of care over a 1-year time horizon, a time horizon frequently used in the cost-effectiveness analysis of cholecystectomy modalities [14-18]. We selected a decision tree model, as this approach was able to appropriately represent the associated costs and health outcomes, including potential complications, and is consistent with prior literature in the field [14-18]. Under each care model, the procedure was either completed laparoscopically or converted to open cholecystectomy. Patients then experienced either no postoperative complications, minor postoperative complications, major postoperative complications, or acute mortality [11]. The branch probabilities in the decision tree model, extracted from the existing literature [11], estimated the likelihood of a patient reaching these endpoints. Patients without postoperative complications and those with Clavien-Dindo grade 1/2 complications [19] were grouped together in the no postoperative complication group because they have similar lengths of stay [11]. Minor postoperative complications were considered surgery outcomes of Clavien-Dindo grade 3 complications [19], reserved for those requiring a procedure after a complication [11]. Major postoperative complications were those with any Clavien-Dindo grade 4 complications [19], described as any life-threatening complication requiring an intensive level of care [11]. Acute mortality was defined as the death of the patient within 30 days postoperatively (Figure 2).

### Model Variables: Probabilities

Probabilities for each decision tree branch used in the base case and sensitivity analyses are presented in Table 1. The probabilities of clinical events were extracted from published literature, with sources indicated in Table 1, including probabilities for converting to open cholecystectomies and minor or major postoperative complications.

**Table 1.** Model parameters extracted from the published literature.

Parameter	Base case estimate	Range [SD]^a^	Source
Model parameters under the conventional model of care			
Decision tree branch probabilities			
Laparoscopic cholecystectomy without conversion to open	0.97	0.96‐0.98 [0.18]	[14,20]
No complications	0.94	0.90‐0.98 [0.18]	[14,20-22]
Minor complications	0.06	0.04‐0.09 [0.84]	[14,20-22]
Laparoscopic cholecystectomy with conversion to open	0.03	0.02‐0.05 [0.25]	[14,20]
No complications	0.88	0.84‐0.94 [0.18]	[14,20]
Minor complications	0.08	0.04‐0.16 [1.83]	[14,23]
Major complications	0.01	0.00‐0.06 [0.94]	[14,23]
Acute mortality	0.02	0.01‐0.08 [1.15]	[14,23]
Utilities			
Laparoscopic cholecystectomy without conversion to open			
No complications	0.98	0.93‐0.99 [0.02]	[14,24]
Minor complications	0.97	0.92‐0.99 [0.02]	[14,24]
Laparoscopic cholecystectomy with conversion to open			
No complications	0.87	0.82‐0.92 [0.1]	[14,24]
Minor complications	0.85	0.78‐0.90 [0.1]	[14,24]
Major complications	0.82	0.77‐0.87 [0.1]	[14,24]
Acute mortality	0	0	
Bed days			
Laparoscopic cholecystectomy without conversion to open			
No complications	3	1‐5	[14,20-22]
Minor complications	5	2‐8	^[14,20-22]^
Laparoscopic cholecystectomy with conversion to open			
No complications	4	2‐8	^[14,22,25,26]^
Minor complications	7	3‐11	^[14,22,25,26]^
Major complications	8	4‐15	^[14,22,25,26]^
Acute mortality	8	1‐15	^[14,22,25,26]^
Health care costs (2023 AU $)^b^			
Laparoscopic cholecystectomy without conversion to open			
No complications	$11,300	$5,650 – $16,950 [$5,650]	[27]
Minor complications	$11,300	$5,650 – $16,950 [$5,650]	[27]
Laparoscopic cholecystectomy with conversion to open			
No complications	$17,238	$8,619 – $25,857 [$8,619]	[27]
Minor complications	$17,238	$8,619 – $25,857 [$8,619]	[27]
Major complications	$43,575	$21,787 – $65,362 [$21,787]	[27]
Acute mortality	$38,149	$19,074 – $57,223 [$19,074]	[27]
Model parameters under the acute day-surgery model of care			
Decision tree branch probabilities			
Laparoscopic cholecystectomy without conversion to open	0.97	0.96‐0.98 [0.18]	[14,20]
No complications	0.94	0.90‐0.98 [0.18]	[14,20-22]
Minor complications	0.06	0.04‐0.09 [0.84]	[14,20-22]
Laparoscopic cholecystectomy with conversion to open	0.03	0.02‐0.05 [0.25]	[14,20]
No complications	0.88	0.84‐0.94 [0.18]	[14,20]
Minor complications	0.08	0.04‐0.16 [1.83]	[14,23]
Major complications	0.01	0.00‐0.06 [0.94]	[14,23]
Acute mortality	0.02	0.01‐0.08 [1.15]	[14,23]
Utilities			
Laparoscopic cholecystectomy without conversion to open			
No complications	0.98	0.93‐0.99 [0.02]	[14,24]
Minor complications	0.97	0.92‐0.99 [0.02]	[14,24]
Laparoscopic cholecystectomy with conversion to open			
No complications	0.87	0.82‐0.92 [0.1]	[14,24]
Minor complications	0.85	0.78‐0.90 [0.1]	[14,24]
Major complications	0.82	0.77‐0.87 [0.1]	[14,24]
Acute mortality	0	0	
Bed days			
Laparoscopic cholecystectomy without conversion to open			
No complications	1	1‐2	[14,20-22]
Minor complications	5	2‐8	^[14,20-22]^
Laparoscopic cholecystectomy with conversion to open			
No complications	4	2‐8	^[14,22,25,26]^
Minor complications	7	3‐11	^[14,22,25,26]^
Major complications	8	4‐15	^[14,22,25,26]^
Acute mortality	8	1‐15	^[14,22,25,26]^
Health care costs (2023 AU $)			
Laparoscopic cholecystectomy without conversion to open			
No complications	$8,188	$4,094 – $12,282 [$4,094]	[27]
Minor complications	$11,300	$5,650 – $16,950 [$5,650]	[27]
Laparoscopic cholecystectomy with conversion to open			
No complications	$17,238	$8,619 – $25,857 [$8,619]	[27]
Minor complications	$17,238	$8,619 – $25,857 [$8,619]	[27]
Major complications	$43,575	$21,787 – $65,362 [$21,787]	[27]
Acute mortality	$38,149	$19,074 – $57,223 [$19,074]	[27]
Remote patient monitoring	$1,556^c^	$500 – $5000 [$778]	[28]

a The range values were used in the one-way sensitivity analysis whilst the SD values were used in the probabilistic sensitivity analysis.

b As all costs were originally calculated in Australian dollars, a currency exchange rate of AU $1 = US $0.66 is applicable.

c We assumed that the remote patient monitoring cost component would not exceed $3112 per patient ($11,300-$8188), which was the cost reduction from converting conventional inpatient laparoscopic cholecystectomies to same-day procedures. The base case cost for remote patient monitoring was set at 50% of the cost reduction ($3,112 × 0.5 = $1556), equivalent to $518.60 per person-day for remotely monitoring a patient for 3 days.

### Model Variables: Costs

The cost information for cholecystectomies was extracted from the Independent Hospital and Aged Care Pricing Authority [27]. This included information on laparoscopic cholecystectomies with varying degrees of complexity, ranging from minor to major, as well as for open cholecystectomies. The same cost for surgeries was applied for cases with no postoperative complications or minor postoperative complications only, but higher rates were applied for major complications. For laparoscopic cholecystectomies converted to open, we conservatively used the costs of open cholecystectomies. There was no published Australian cost information on cholecystectomies under the new model of care, so we used information from the literature to estimate these costs. Specifically, Manzia et al [28] found that conducting conventional inpatient laparoscopic cholecystectomies as same-day procedures reduced the cost per procedure by 1.38 times. We applied this ratio to local estimates for costs for laparoscopic cholecystectomies with no postoperative complications and parameterized this for the new same-day care model (Table 1). Our assumptions for other model parameterization were consistent with existing literature [7,9,29] that has indicated that hospital-in-the-home type care with remote patient monitoring is typically cheaper than the inpatient care alternative for uncomplicated surgical patients (Table 1). Remote patient monitoring costs included costs of equipping, establishing, and staffing the remote monitoring program for each patient case. Since all costs were originally calculated in Australian dollars, a currency exchange rate of AU $1 = US $0.66 is applicable.

### Model Variables: Bed Days and Health-Related Quality of Life

An important motivation for considering laparoscopic cholecystectomies as same-day procedures stems from hospital operational efficiency innovation intended to free up inpatient bed capacity and potentially reduce waiting times for surgeries [7-9]. Consequently, our main outcome was inpatient bed days saved with the new model of care. Representation of health-related quality of life outcomes in this modeling was also considered important to clinicians and health care administrators contributing to this study as investigators or care model informants. It is common for modeling studies to apply literature-informed estimates for quality-adjusted life year (QALY)-related parameterization, and we were able to draw on literature to assign parameter values for health states represented in the model (Figure 1). However, it was challenging to accommodate potential between-care model differences in QALYs due to a lack of comparative effectiveness studies examining potential differences between the two care models represented in this study. On one hand, it might have been considered reasonable to expect that less time waiting in hospital for surgery, and fewer days in hospital overall to have a small beneficial effect on patients’ health-related quality of life and thus assume favorable QALY parameterization in favor of the new care model. On the other hand, given that most patients will have relatively short lengths of stay in hospital in both care scenarios and that health-related quality of life is likely to primarily be influenced by factors unrelated to the care model, it may also be reasonable to assume that we would not detect a difference in QALY levels attributable to the new model of care. Therefore, in the absence of comparative effectiveness evidence for QALY effects, we adopted the more conservative approach and assumed the same QALY point estimates for both models of care (Table 1) for the present study, but parametrized considerable uncertainty to reflect and highlight the current lack of empirical QALY estimates. While this approach may be considered conservative and likely to produce indeterminate QALY findings, we thought it was appropriate to highlight this uncertainty and the importance of including patient-reported outcomes in future prospective comparative effectiveness studies in this field whether randomized trials or prospective quasi-experimental studies.

### Cost-Effectiveness/Utility Analyses

The primary analysis ascertained the cost-effectiveness of the new acute day-surgery model of care compared to the conventional model of care, using the net incremental benefits approach with the outcome of the number of bed days saved. Net monetary benefit (NMB) estimates were informed by a willingness-to-pay (WTP) threshold derived from a recent local publication [30] originating from New South Wales, Australia. That study estimated the average inpatient cost for patients who received laparoscopic cholecystectomy was AU $3873 per day in 2019 [30], which, if inflated [31] to 2023 values, represents approximately AU $4522. We, therefore, adopted a slightly more conservative WTP threshold of AU $4000 per inpatient day. The model of care that generated the highest incremental net benefits was considered the most cost-effective. The secondary analysis used QALYs as the outcome, and the treatment strategy that generated the highest QALYs, without exceeding an ICER of AU $50,000/QALY was the most cost-effective. We selected the WTP threshold of AU $50,000/QALY because it is the most used value in current health care cost-utility analysis in Australia. The conventional model of care was used as the reference group for all comparisons of costs and outcomes.

We followed the CHEERS guidelines for reporting economic evaluations (Checklist 1).

### Sensitivity Analysis

We conducted several sensitivity analyses to explore how the base case results responded to uncertainties in the parameters. First, we conducted one-way sensitivity analyses to examine the effects of all model parameters on the base case results. As we found no specific cost information on postoperative laparoscopic cholecystectomy remote patient monitoring, we explored broad ranges for these costs. For example, one study [32] reported the cost of remote patient monitoring after same-day laparoscopic sleeve gastrectomy at US $3816 per day for all patients (n=20) monitored by the service, while another study [33] reported the average per person-day cost of US $24 for a remote patient monitoring program for post-discharge management of type 2 diabetes. Our one-way sensitivity analysis for remote patient monitoring cost ranged between AU $166 and AU $1,666 per person-day. We used a tornado diagram to summarize the results of the one-way sensitivity analysis.

Furthermore, using estimated distributions on each model parameter, we performed a probabilistic sensitivity analysis to assess uncertainty in the model results using a Monte Carlo simulation with 5000 samples. We simulated the decision tree branch probabilities and QALYs from beta distributions, bed days from the program evaluation review technique (PERT) distributions, and costs from gamma distributions (Table 1). We used the range of uncertainties extracted from the literature. In cases where no range of uncertainties was reported, we allowed parameters to vary by ±50% of the index value.

### Ethical Considerations

This economic modeling study did not require review by the Queensland University of Technology (QUT) Human Research Ethics Committee. In accordance with QUT’s ethics review policy and the National Statement on Ethical Conduct in Human Research (Section 5.1.22), research that involves only the use of existing, publicly available, non-identifiable data is exempt from ethics review. The data used in this study were obtained exclusively from published sources and contained no identifiable personal information.

## Results

### Base Case

The base case results over a 1-year time horizon indicated that the new acute day-surgery model of care was dominant compared to the conventional model of care considering the main outcome of bed days saved. Specifically, the new model of care saved 1.7 days per patient (3.2 days for the conventional model of care vs 1.5 days for the acute day-surgery model of care) and lowered health care costs by AU $1416 per patient (AU $11,509 for the conventional model of care vs AU $10,093 for the acute day-surgery model of care). This translates to an incremental NMB of AU $8096 per case at the adopted WTP threshold.

Regarding the QALY outcome, the acute day-surgery model of care was marginally cost-effective compared to the conventional model of care. Both models were similar in effectiveness but had lower health care costs for the new care model (AU $11,509 for the conventional model of care vs AU $10,093 for the acute day-surgery model of care).

### Sensitivity Analysis Results

**Figure 3.**
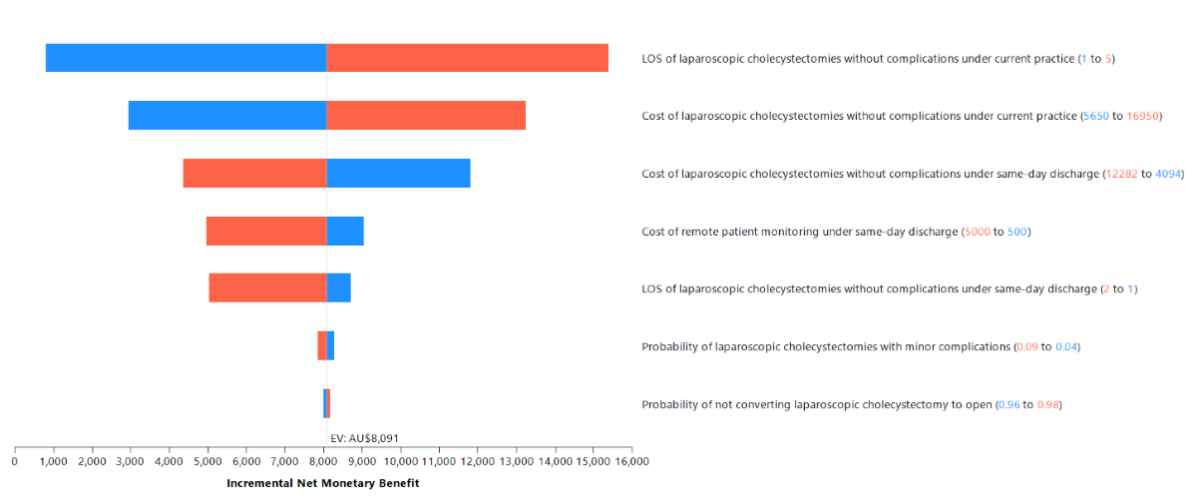
Tornado diagram (willingness-to-pay: AU $4,000). This tornado diagram ranks parameters by their influence on the net monetary benefit by varying each parameter across plausible ranges while holding others constant. The horizontal bars represent the range of results generated by varying each parameter from its minimum to maximum value. Parameters with the greatest impact appear at the top of the diagram, while those with minimal influence are shown at the bottom. The center line indicates the base-case outcome, and the left and right ends of each bar correspond to the outcome values at the lower and upper bounds of the parameter range, respectively. LOS: length of stay. Since all costs were originally calculated in Australian dollars, a currency exchange rate of AU $1 = US $0.66 is applicable.

The deterministic one-way sensitivity analysis results are summarized in the tornado diagram in Figure 3. Findings indicated that the incremental NMB remained generally robust to the ranges of model input parameters explored. Of the 23 model parameters, only 7 had some sensitivity impact on the incremental NMB. The model was most sensitive to the length of stay parameterization for cholecystectomies without complications under the conventional model of care.

**Figure 4.**
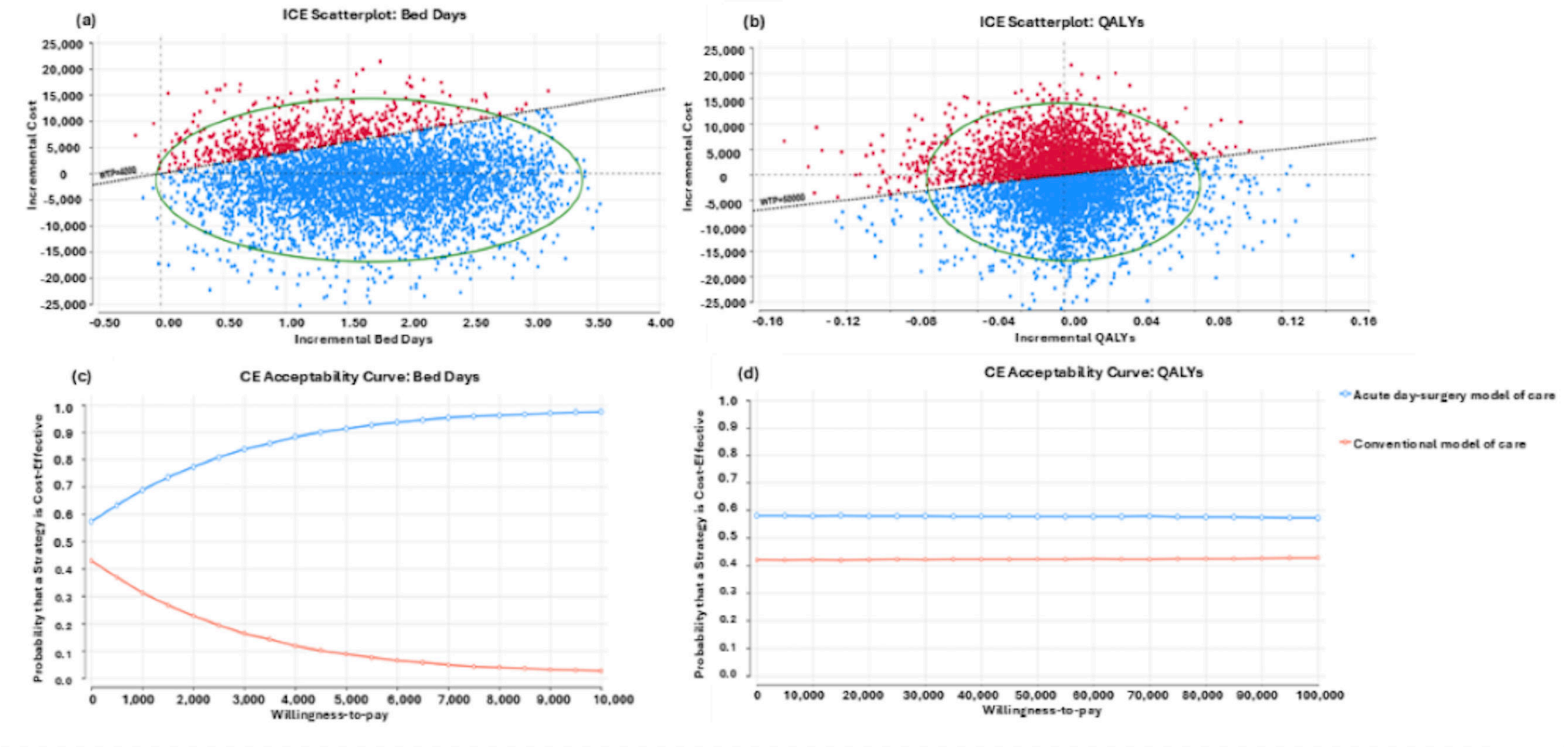
Results of the probabilistic sensitivity analysis presented in the cost-effectiveness planes for bed days (A) and quality-adjusted life years (QALYs) (B), as well as cost-effectiveness acceptability curves for bed days (C) and QALYs (D).

The results of the probabilistic sensitivity analyses are presented in Figure 4. In large proportions of the 5000 re-samples, the new acute day-surgery model of care was either dominant or cost-effective (Figure 4A). There was an 89% probability that the new same-day care model would be cost-effective compared to the conventional model of care at a WTP threshold of AU $4,000 per acute surgical bed day saved (Figure 4C). Similarly, regarding the QALY outcome, the probabilistic sensitivity analysis results showed that the new acute day-surgery model of care was more likely to be cost-effective compared to the conventional model of care (Figure 4B), with a 57% probability of being cost-effective at a WTP threshold of AU $50,000 per QALY.

## Discussion

### Principal Results

This study presents the first cost-effectiveness analysis comparing a dedicated acute day-surgery model of care supported by virtual care with remote patient monitoring to a conventional inpatient-centric model of care for acute cases of cholecystitis. The results indicate that the dedicated acute day-surgery care model with remote patient monitoring was likely to be dominant (ie, saves bed days and incurs lower health care costs). Regarding QALYs, the new same-day discharge care model may be considered marginally cost-effective compared to the conventional inpatient-centric practice, which reflected our conservative approach of not assuming potential improvements in QALYs that may be associated with less time in hospital. Both the one-way and probabilistic sensitivity analyses indicated that findings remained robust across potential model parameter ranges.

### Comparison With Prior Work

Our study is aligned with the latest developments in the field. A systematic review of discordant meta-analysis [4] recommended early laparoscopic cholecystectomy over delayed laparoscopic cholecystectomy. One strategy often suggested to expedite surgery for patients with acute biliary disease is to dedicate theater resources and surgical expertise to same-day discharge protocols for laparoscopic cholecystectomies [8]. A single hospital visit pathway for day-case laparoscopic cholecystectomy has been trialed and found to be feasible, safe, and acceptable for patients with symptomatic gallstone disease [34]. This is consistent with other literature [28] that established ambulatory laparoscopic cholecystectomy as both safe and cost-effective. A recent systematic review [10] has also reported an “optimal” inclusion criterion for successful same-day cholecystectomies in Australia. It is also noteworthy that there have been previous favorable trials of protocols offering patients same-day laparoscopic sleeve gastrectomy supported by remote patient monitoring [32,35], and an acute day-only surgery program for abscess drainage [12].

Recent systematic reviews [7,9,29] including studies from other clinical populations have concluded virtual wards, hospital-in-the-home, and other remote patient monitoring interventions had positive impacts on patient safety, adherence, patients’ mobility, functional statuses, and cost-related outcomes. However, investigations of the impact of remote patient monitoring on quality-of-life indicators remain inconclusive [7,9,29]. Other studies examining safety and feasibility of assessing and counseling patients for laparoscopic cholecystectomy remotely without a physical encounter [36] support the use of remote follow-up [37,38]. Similarly, a virtual clinic for post-operative patients who underwent laparoscopic cholecystectomy improved clinic efficiency [39].

While the remote patient monitoring evidence base is growing, evidence regarding the cost and cost-effectiveness implications of these innovations is lagging. A scoping review [40] considering economic impacts concluded that telehealth provides positive patient benefits and improves productivity for many services, but does not routinely reduce costs for health care systems. A systematic review of economic evaluations of remote patient monitoring interventions for chronic diseases found that remote patient monitoring interventions were highly cost-effective for hypertension, differed according to disease severity for chronic obstructive pulmonary disease and heart failure, and had limited economic evidence among patients with diabetes [41]. Our results extend this evidence base by indicating that bundling acute day surgeries for cholecystitis with virtual care, including remote monitoring, is likely to be cost-effective.

### Limitations

This study has several limitations. Comparative-effectiveness evidence for the impact of remote patient monitoring use on QALYs in this patient group is lacking, and it may be important to update these modeling results as new evidence is reported. Randomized trials examining the impact of novel care models, including remote-patient monitoring, would be informative in this regard. Furthermore, we did not account for procedural complications such as incisional hernia because they are relatively rare, and we considered them unlikely to impact findings [14]. Similarly, the lack of granular public domain cost information for pre- and post-operative remote patient monitoring meant that we applied a wide range of potential costs, but fortunately, the sensitivity analyses indicated results were robust to this uncertainty.

### Conclusions

The acute day-surgery model of care supported by remote patient monitoring was dominant. It is likely to save bed days and incur lower health care costs compared to the conventional inpatient-centric care model for acute cholecystitis cases.

## Supplementary material

10.2196/76807 Checklist 1 CHEERS 2022 Checklist

## Abbreviations

ASA: American Society of Anaesthesiologists
ED: emergency department
NMB: net monetary benefit
QALY: quality-adjusted life year
QUT: Queensland University of Technology
WTP: willingness-to-pay
